# Enhancing Clinical Relevance of Pretrained Language Models Through Integration of External Knowledge: Case Study on Cardiovascular Diagnosis From Electronic Health Records

**DOI:** 10.2196/56932

**Published:** 2024-08-06

**Authors:** Qiuhao Lu, Andrew Wen, Thien Nguyen, Hongfang Liu

**Affiliations:** 1 McWilliams School of Biomedical Informatics University of Texas Health Science Center Houston, TX United States; 2 Department of AI and Informatics Mayo Clinic Rochester, MN United States; 3 Department of Computer Science University of Oregon Eugene, OR United States

**Keywords:** knowledge integration, pre-trained language models, physician reasoning, adapters, physician, physicians, electronic health record, electronic health records, EHR, healthcare, heterogeneous, healthcare institution, healthcare institutions, proprietary information, healthcare data, methodology, text classification, data privacy, medical knowledge

## Abstract

**Background:**

Despite their growing use in health care, pretrained language models (PLMs) often lack clinical relevance due to insufficient domain expertise and poor interpretability. A key strategy to overcome these challenges is integrating external knowledge into PLMs, enhancing their adaptability and clinical usefulness. Current biomedical knowledge graphs like UMLS (Unified Medical Language System), SNOMED CT (Systematized Medical Nomenclature for Medicine–Clinical Terminology), and HPO (Human Phenotype Ontology), while comprehensive, fail to effectively connect general biomedical knowledge with physician insights. There is an equally important need for a model that integrates diverse knowledge in a way that is both unified and compartmentalized. This approach not only addresses the heterogeneous nature of domain knowledge but also recognizes the unique data and knowledge repositories of individual health care institutions, necessitating careful and respectful management of proprietary information.

**Objective:**

This study aimed to enhance the clinical relevance and interpretability of PLMs by integrating external knowledge in a manner that respects the diversity and proprietary nature of health care data. We hypothesize that domain knowledge, when captured and distributed as stand-alone modules, can be effectively reintegrated into PLMs to significantly improve their adaptability and utility in clinical settings.

**Methods:**

We demonstrate that through adapters, small and lightweight neural networks that enable the integration of extra information without full model fine-tuning, we can inject diverse sources of external domain knowledge into language models and improve the overall performance with an increased level of interpretability. As a practical application of this methodology, we introduce a novel task, structured as a case study, that endeavors to capture physician knowledge in assigning cardiovascular diagnoses from clinical narratives, where we extract diagnosis-comment pairs from electronic health records (EHRs) and cast the problem as text classification.

**Results:**

The study demonstrates that integrating domain knowledge into PLMs significantly improves their performance. While improvements with ClinicalBERT are more modest, likely due to its pretraining on clinical texts, BERT (bidirectional encoder representations from transformer) equipped with knowledge adapters surprisingly matches or exceeds ClinicalBERT in several metrics. This underscores the effectiveness of knowledge adapters and highlights their potential in settings with strict data privacy constraints. This approach also increases the level of interpretability of these models in a clinical context, which enhances our ability to precisely identify and apply the most relevant domain knowledge for specific tasks, thereby optimizing the model’s performance and tailoring it to meet specific clinical needs.

**Conclusions:**

This research provides a basis for creating health knowledge graphs infused with physician knowledge, marking a significant step forward for PLMs in health care. Notably, the model balances integrating knowledge both comprehensively and selectively, addressing the heterogeneous nature of medical knowledge and the privacy needs of health care institutions.

## Introduction

### Background

In recent years, pretrained language models (PLMs) have revolutionized many areas of natural language processing (NLP), demonstrating proficiency in handling a broad spectrum of general-domain text tasks. However, their performance declines when confronted with specialized domains, such as health care, as clinical text often presents unique linguistic characteristics and semantics that differ from standard language [1,2]. The extensive proliferation of electronic health records (EHRs) further underscores the gap, highlighting the demand for domain-specific methods in PLMs.

Although there are domain-specific PLMs designed by training on large-scale clinical data sets, they often fail to capture the depth and breadth of knowledge scattered across diverse biomedical sources [3]. This limitation calls for an approach that integrates specific domain knowledge into PLMs, enhancing their effectiveness and accuracy in specialized contexts.

To address this need, we propose a dual strategy—the strategic incorporation of external knowledge from diverse sources in a unified yet compartmentalized manner. Biomedical domain knowledge is inherently heterogeneous and stored in a variety of formats. A unified approach that simultaneously incorporates various knowledge sources is essential to manage this diversity. Traditional methods of sequential training with new knowledge sources are inefficient and risk losing previously integrated knowledge due to continuous model parameter adjustments. A unified model overcomes these challenges by integrating diverse knowledge without the need for repeated, individualized retraining.

Furthermore, the broad diversity of domain knowledge sources, each relevant to different tasks in its own way, underscores the need for a compartmentalized approach. This strategy allows for the selective integration of the most relevant knowledge, avoiding information overload. In addition, given that each institution manages its own proprietary repository of data and knowledge, often governed by protected health information (PHI) regulations, a method that potentially respects institutional boundaries is desirable. This could enable an institution to freely choose to equip a widely shareable foundational model with its particular data, thereby enabling an adaptable and compliant framework that can cater to diverse institutional needs without compromising data privacy and security.

Building on this rationale, we introduce a specific case study in the cardiovascular domain to demonstrate our approach. This involves extracting diagnosis-comment pairs from EHRs and approaching the problem through text classification, predicting diagnoses based on physician comments. Essentially, we take PLMs with a linear head on top as the foundational prediction model and fine-tune them on this specific task, optimizing it to better capture the specialized knowledge and clinical terminologies present in physician comments within the cardiovascular domain.

As most clinical PLMs, such as clinical bidirectional encoder representations from transformer (ClinicalBERT) [4], are primarily trained on large-scale free texts and lack integration with structured domain knowledge, they often demonstrate suboptimal performance in knowledge-driven tasks [5-7]. To address this limitation, we incorporate the Diverse Adapters for Knowledge Integration (DAKI) framework [6] for knowledge infusion, which integrates domain knowledge adaptively from multiple sources. More specifically, we train 3 distinct adapters, each tailored to encapsulate domain knowledge from a specific source, that are (1) the Unified Medical Language System (UMLS) Metathesaurus, (2) Wikipedia articles, and (3) semantic grouping information for biomedical concepts. This approach effectively augments PLMs, enhancing their performance within the clinical context. The adapter-enhanced PLMs retain a unified utility, functioning as standard PLMs, while simultaneously featuring a compartmentalized structure, where adapters are incorporated in a plug-and-play manner, ensuring flexibility and transferability. The contributions can be summarized as follows: (1) we propose a novel task aimed at capturing physician knowledge in the cardiovascular domain through text classification of diagnosis-comment pairs from EHRs. The encouraging performance of our models on this task validates its feasibility, demonstrating the potential of PLMs in capturing medical insights. (2) Upon integrating domain knowledge through the DAKI framework, the models not only exhibit enhanced performance but also an increased level of interpretability, where we can closely examine and clarify which external domain knowledge is activated during tasks. Such interpretability could further enable the identification of vital knowledge pieces, refine the fine-tuning of models for particular tasks, and assist in adjusting the applied domain knowledge to be more task-specific. (3) The domain knowledge demonstrates transferability when injecting respective adapters into different PLMs, where pretrained knowledge adapters also prove effective when equipped with other, previously unseen PLMs. This highlights the potential for heterogeneous knowledge infusion while considering institutional boundaries, laying a foundational step toward the development of health knowledge graphs enriched with physician knowledge.

### Related Work

Patient diagnosis prediction is a challenging task due to the complex and knowledge-intensive nature of this field. Most existing studies heavily rely on codified, numerical, or time-series features of patients, where significant features are manually selected as input to downstream machine learning models. Franz et al [8] extracted all numerical observations from MIMIC-III (Medical Information Mart for Intensive Care III) data set [9], for example, vital sign measures and lab results, and fed them as input into a 4-layer neural network (1 convolutional neural network [10] layer spanning across the time dimension followed by 3 fully connected layers) for multiclass classification. Zoabi et al [11] selected a set of features including sex, age, symptoms (cough, fever, sore throat, shortness of breath, and headache), and known contact as input and fed them into a gradient-boosting machine model to track COVID-19. Meanwhile, with the rapid growth of NLP techniques, researchers have been exploring the clinical notes of EHRs for a wide variety of clinically relevant tasks, including diagnosis prediction. For example, Franz et al [8] fine-tuned ClinicalBERT [12] for the prediction and significantly outperformed their numerical method. Another line of research aimed to leverage the multimodality of EHRs as there exists rich structural information within EHRs, for example, the interactions among users, symptoms, and diseases [13], where these interactions are captured through encoding EHRs through graph neural networks [14,15]. The task of this work differs from the aforementioned studies in that we only use a single piece of physician comment as input, and instead of pushing state-of-the-art predictive performance, we try to understand the insight of a physician by capturing their reasoning on the diagnosis.

While PLMs excel on general-domain text, their performance over domain-specific text is relatively poor due to domain shift [2]. In the last few years, several domain-specific PLMs have been proposed to mitigate the issue, for example, BioBERT [16], ClinicalBERT [12], ClinicalBERT [4], PubMedBERT [17], ClinicalT5 [18], etc. Despite their specificity, training these models demands significant time and resources. Moreover, recent findings indicate that even these specialized models can struggle in certain scenarios, particularly when reliable knowledge retrieval is essential for complex domain-specific reasoning [3].

Beyond acquiring domain knowledge through pretraining, a distinct research trajectory emphasizes knowledge infusion, wherein domain knowledge is intentionally injected into language models [6,7,19-23]. Typically, this involves adding an auxiliary training objective driven by knowledge. This approach facilitates additional pretraining or fine-tuning of existing models, thereby cutting down on training expenses, though it can still demand significant resources. For instance, Wang et al [7] jointly optimized language modeling with a knowledge embedding objective. Zhang et al [23] fused PLMs with graph neural networks through layered modality interactions, enabling bidirectional information flow for enhanced reasoning in question-answering tasks. Our choice to use DAKI [6] for knowledge infusion is motivated by 3 principal reasons, that are (1) the framework integrates domain knowledge of varied sources and formats, which reflects the heterogeneous nature of the domain knowledge; (2) focusing on training adapters, instead of the entire language model, presents a more sustainable and efficient approach; and (3) the knowledge adapters are integrated in a plug-and-play manner that increases both flexibility and interpretability.

### Proposed Task Design

#### Data Collection and Structure

The experiment was conducted using clinical notes generated by the Mayo Clinic Rochester Campus between January 1 and December 31, 2015, corresponding to roughly 5 million documents. Specifically, we extracted the problem entries from the Impression/Report/Plan (IRP) section in the clinical note as it contained a diagnostic problem list that was used to summarize the main findings [24]. The entries are recorded as numbered items and each item is a textual description of the diagnosis followed by a physician comment detailing their reasoning for giving a diagnosis. We then convert them into <entity, comment> pairs by mapping the textual descriptions of diagnosis to entities and associated UMLS concept unique identifiers (CUIs) using SciSpacy [25]. We specifically perform entity linking for diseases and syndromes, in light of the observation that medical interests arise primarily around symptoms and problems [26]. After filtering to only clinical narratives generated in the Department of Cardiovascular Medicine and removing unrecognized or unlinkable texts, 174,980 valid pairs were generated corresponding to 30,240 patients. We then split the data into 10 folds where 8 folds for training, 1 fold for development, and 1 fold for testing were at the patient level.

#### Task Objective and Metrics

The task is cast as a multiclass text classification problem, that is, to predict the assigned diagnosis (entity) from a physician’s comment detailing their reasoning for assigning a diagnosis. As most (linked) entities occur only once in the prepared data set, we use the most frequent top 50 entities as the targets for all experiments in this study. For instance, the top 10 most frequent entities that appear in the training set are “hypertensive disease,” “hyperlipidemia,” “sleep apnea, obstructive,” “atrial fibrillation,” “coronary arteriosclerosis,” “hypothyroidism,” “diabetes mellitus, non-insulin-dependent,” “gastroesophageal reflux disease,” “chronic kidney diseases,” and “dyslipidemias.” We use the top-k (k=1,3,5,10) accuracy classification score as the evaluation metric, which computes the number of times where the correct label is among the top-k labels predicted (ranked by predicted scores).

## Methods

We consider 2 prediction models in this study, that are, the PLMs and those equipped with DAKI [6].

### Foundational Models

For the foundational prediction models, we used BERT-base-uncased [27], ALBERT-xxlarge-v2 [28], and ClinicalBERT-base [4] to cover base or large, and general or specific domain variants. Essentially, we encode the physician comment with the models and feed the average pooled representations into a linear layer for prediction. The model is fine-tuned by optimizing a cross-entropy loss.

### Models Equipped With DAKI

**Figure 1:**
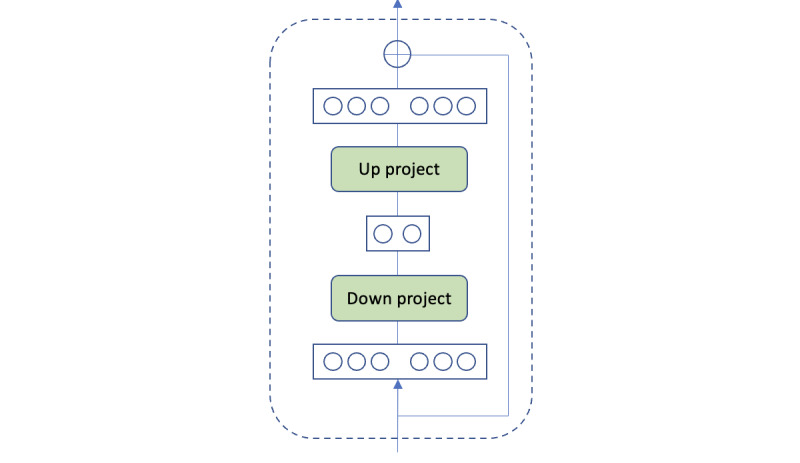
Adapter module, for example, a bottleneck feed-forward network with a residual connection.

**Figure 2:**
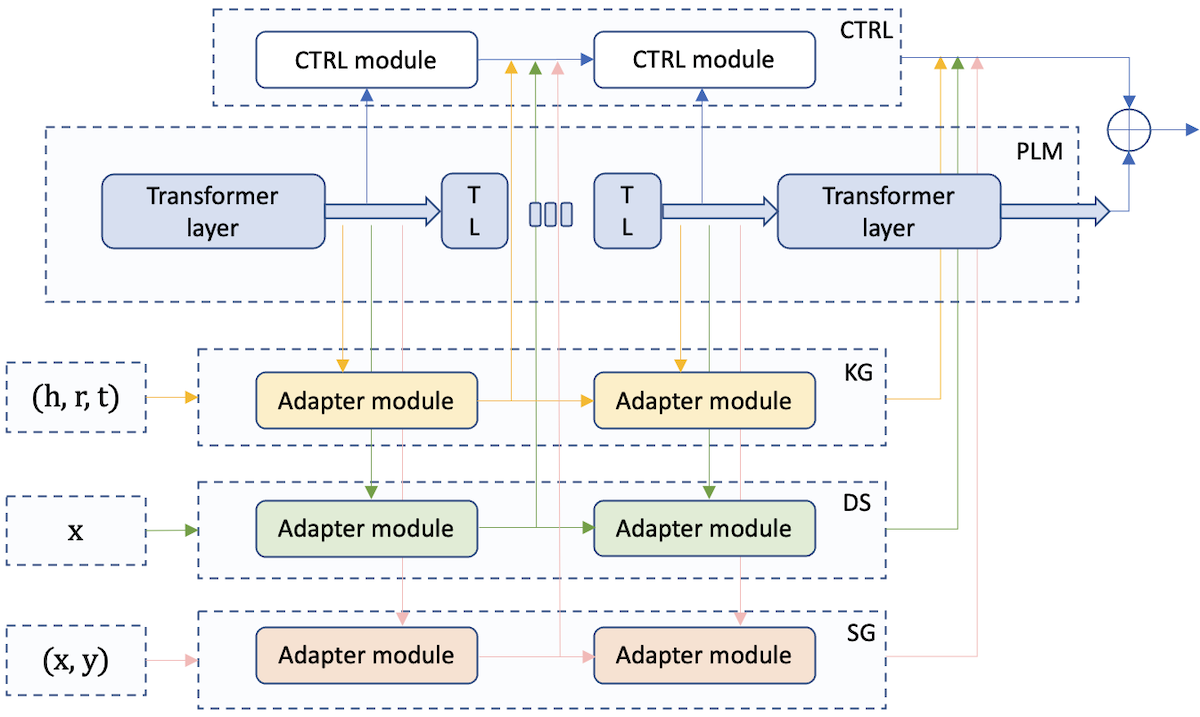
Architecture of DAKI [6]. CTRL: knowledge controller; DAKI: Diverse Adapters for Knowledge Integration; DS: disease adapter; h, r, t: head, relation, tail; KG: knowledge graph adapter; PLM: pretrained language model; SG: semantic grouping adapter; TL: transformer layer.

To facilitate prediction on clinical text, we leverage a novel framework that incorporates DAKI into PLMs. The adapter in this framework is a small bottleneck feed-forward network with a residual connection that is placed within PLMs, as illustrated in Figure 1. One can also incorporate a more advanced adapter structure, such as the LoRA (low-rank) adapter [29]. This framework consists of 3 major components, which are the base PLM, pretrained knowledge-specific adapters, and the knowledge controller (CTRL) that adaptively activates the adapters, as illustrated in Figure 2. Generally, when pretraining a knowledge adapter, the parameters of the base PLM are frozen, and only the adapter is optimized. In this way, we inject specific knowledge into an adapter. By equipping PLMs with adapters, one can inject domain knowledge into the models without touching the original parameters of PLMs, enhancing their representation capabilities on domain-specific text. Essentially, a knowledge adapter is independently pretrained to encode domain knowledge, and the trained adapters are then plugged into DAKI for downstream fine-tuning, where the knowledge adapters are adaptively activated by the knowledge controller. Therefore, the usage of DAKI is simple and straightforward as the output can be considered as the last hidden states of a PLM.

We use the best version of ALBERT (ie, ALBERT-xxlarge-v2 [28]) as the base PLM for adapter pretraining. In this study, we incorporate 3 clinically relevant knowledge adapters that integrate disparate domain knowledge from the UMLS Metathesaurus (knowledge graph adapter [KG]), the Wikipedia articles for diseases (disease adapter [DS]), and the semantic groupings (semantic grouping adapter [SG]). More specifically, the KG captures relational patterns within medical entities using the UMLS Metathesaurus. It is trained on triples from UMLS, treated as textual sequences, to predict the plausibility of these relational statements. For the DS, disease-related textual descriptions are sourced from Wikipedia, with the training process focusing on inferring disease names through masked language modeling, enhancing the model’s grasp on disease contexts. The SG uses UMLS semantic groupings to predict the categorization of medical concepts, leveraging textual definitions to understand and classify medical concepts into coherent groupings. Essentially, we try to enforce the KG to capture the relationships between medical entities, the DS to help PLMs understand the definitions and contexts for diseases, and the SG to maintain semantic coherence within a categorization group. We refer the readers to our previous work for a more detailed treatment of the architecture and training objectives of DAKI [6].

We equip the 3 foundational models, that is, BERT, ALBERT, and ClinicalBERT, with DAKI, respectively, and enable all the previously trained adapters within the framework for the experiments. Likewise, we encode the physician comments with the DAKI models and feed the average pooled representations to a linear layer for prediction.

### Ethical Considerations

We used the Mayo Clinic IRP data, and this study was approved by the Mayo Clinic Institutional Review Board (#20-001137) for human participants research. The data were not anonymous. No compensation was offered to participants in the study. Due to the presence of private health information in the clinical data set, we do not distribute any recordings or models trained on these recordings. Access to the clinical data is restricted to Mayo Clinic researchers who have the appropriate authorization.

## Results

### Overview

We present the performance of models both with and without DAKI on the test set and development set in Table 1. Generally, all the models are fine-tuned on the development set, and the best epochs of that are selected to report their performance on the test set. The results indicate that the infusion of domain knowledge into PLMs through DAKI consistently boosts their overall performance. Notably, DAKI-ALBERT demonstrates compelling performance gain over ALBERT across all the metrics, compared with the other 2 foundational models, which is almost expected as the adapters are trained with ALBERT as the base PLM. On the other hand, the improvement with ClinicalBERT is comparatively slight, and we hypothesize that this is due to ClinicalBERT’s extensive exposure to clinical text during its pretraining, rendering DAKI-ClinicalBERT less striking.

Another key observation from our results is that DAKI-BERT not only matches but in certain metrics surpasses the performance of ClinicalBERT. This highlights the advantages of incorporating knowledge adapters, particularly given that DAKI-BERT achieves such results without needing extensive and sensitive clinical text corpora. Such transferability of knowledge adapters also indicates a potential for heterogeneous knowledge infusion while respecting institutional boundaries, especially in contexts where each institution possesses its own exclusive data repository due to PHI constraints.

Moreover, considering it is a complex 50-class classification problem, these results are commendably robust. They not only shed light on the feasibility of encapsulating physician reasoning but also highlight the potential of transferable or portable domain knowledge.

**Table 1:** Overall performance.

Data sets and metrics		Test					Development			
		Acc@1^a^	Acc@3	Acc@5	Acc@10	Acc@1		Acc@3	Acc@5	Acc@10
**Without DAKI^b^, %**										
	BERT	48.81	69.7	76.73	85.37	50.56		70.21	76.84	84.92
	ALBERT	48.02	69.35	76.7	85.82	50.47		69.37	76	84.6
	ClinicalBERT	48.49	69.88	77.05	85.96	50.63		70.27	76.87	84.97
**With DAKI, %**										
	BERT	48.2	70.26	77.64	86.38	50.59		70.19	76.77	84.78
	ALBERT	48.32	69.93	77.6	86.3	50.7		70.48	76.82	84.84
	ClinicalBERT	48.73	70.15	77.05	85.94	51.14		69.99	76.45	84.75

^a^Acc@k: the number of times where the correct label is among the top-k labels predicted (ranked by predicted scores).

^b^DAKI: Diverse Adapters for Knowledge Integration.

### Ablation Study

To investigate the influence of each of the knowledge adapters, we conduct an ablation study and show the results in Table 2. We take DAKI-BERT and DAKI-ClinicalBERT for comparison as they have the same number of parameters. We gradually remove the knowledge adapters from the complete setting (ie, all 3 equipped) and this makes 6 conditions, as shown in the table. Essentially for DAKI-BERT, the results of the ablated models demonstrate varying degrees of decline in performance, indicating the necessity of each source of external knowledge. For DAKI-ClinicalBERT, however, the situation is different. When 1 knowledge adapter is removed (ie, KG or DS), the performance gets improved, which is consistent with our conjecture that ClinicalBERT has been exposed to clinical knowledge during pretraining and this weakens the knowledge adapters’ impact. When 2 knowledge adapters are removed, the performance gets decreased at a lower level compared with that of DAKI-BERT, indicating the effectiveness and complementary nature of the knowledge adapters.

**Table 2:** Ablation analysis on the test set.

Ablated model and metrics		DAKI^a^-BERT						DAKI-ClinicalBERT				
		Acc@1^b^	Acc@3	Acc@5	Acc@10	Δ^c^	Acc@1		Acc@3	Acc@5	Acc@10	Δ
**Baseline (no ablation), %**												
	With all	48.2	70.26	77.64	86.38	—^d^	48.73		70.15	77.05	85.94	—
**1 adapter removed, %**												
	KG^e^	48.04	69.57	77.15	85.85	–1.87	48.65		70.16	77.77	86.8	1.50
	DS^f^	47.62	70.51	77.32	85.85	–1.18	48.49		69.87	77.78	86.41	0.67
	SG^g^	48.49	69.91	77.51	86.39	–0.17	48.65		69.49	76.91	86.22	–0.60
**2 adapters removed, %**												
	KG-DS	48.39	69.66	76.77	85.97	–1.68	48.25		69.28	77.32	86.23	–0.80
	KG-SG	48.83	69.93	76.63	85.81	–1.68	48.52		69.59	77.22	86.18	–0.36
	DS-SG	47.55	69.63	76.94	86.06	–2.30	48.03		69.67	77.47	86.39	–0.31

^a^DAKI: Diverse Adapters for Knowledge Integration.

^b^Acc@k: the number of times where the correct label is among the top-k labels predicted (ranked by predicted scores).

^c^Δ: the change of accumulated accuracy scores.

^d^Not applicable.

^e^KG: knowledge graph adapter.

^f^DS: disease adapter.

^g^SG: semantic grouping adapter.

### Analysis

In this section, we want to analyze and answer the following research questions: (1) what knowledge is lacking in the PLMs for the physician reasoning task? (2) How do the models perform on different target diagnoses, that is, at the individual level? (3) How does the knowledge affect the representations at the token level?

### Knowledge Activation

Due to DAKI’s inherent flexibility, we are able to provide a high-level representation of the adapter activations during the inference process. As depicted in Multimedia Appendix 1, we compute the softmax activations across 3 key layers within the encoders of DAKI-BERT (left) and DAKI-ClinicalBERT (right) where the adapters are situated. These activations are then averaged across all test set instances. Notably, the disease knowledge consistently stands out in its importance and activation across these layers, when compared with the other 2 knowledge types. We conjecture that the specificity and relevance of DS to the model’s tasks allow it to have a more significant influence on the encoder’s activation patterns. This reinforces the notion that domain-specific knowledge, particularly when closely aligned with the predictive tasks, is crucial for the model’s decision-making process. The injected knowledge also demonstrates a more pronounced effect on BERT than on ClinicalBERT. This distinction is likely because ClinicalBERT has previously encountered clinical data sets during its pretraining phase, which aligns with the observations detailed in Table 1. The diminished reliance of ClinicalBERT on the knowledge adapters underscores the importance of identifying knowledge that truly complements specific PLMs.

### Impact Pattern

**Figure 3:**
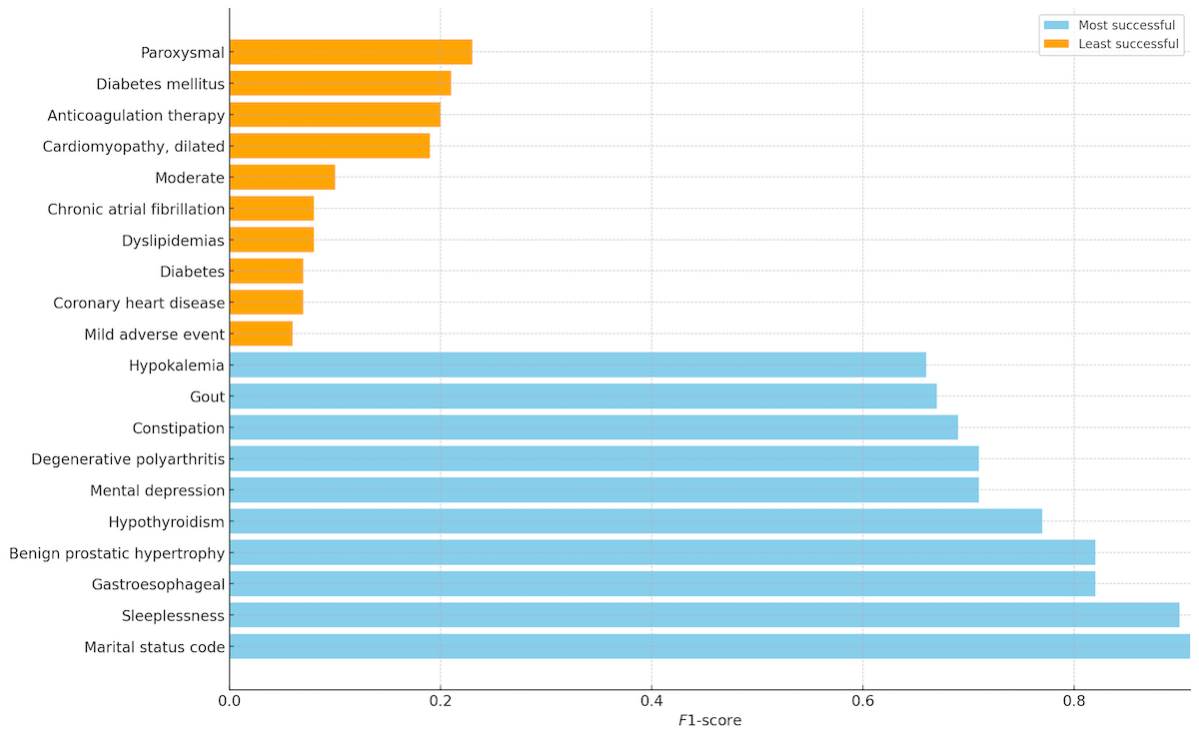
Understanding performance variability: most versus least successful targets.

We also investigate the impact pattern of these knowledge adapters. Essentially, we show the top 10 most and least successful targets in Figure 3. We also observe that the targets with the biggest improvement are among the least successful targets, as shown in Multimedia Appendix 2. The impact is evaluated in terms of the *F*_1_-score.

In general, we believe targets that demand more tests to diagnose are easier to predict, for example, Gout. Such targets might demonstrate more unique textual context in the comment that facilitates the prediction. On the other hand, targets that are easier to diagnose are more challenging for the model to identify. For instance, it makes more sense to diagnose “Diabetes” with “150 mg/dL” than with “blood sugar.” Moreover, we observe that nearly half (ie, 4 out of 10) of the most-impacted targets are among the least successful ones (ie, represented in orange in Multimedia Appendix 2). This pattern underscores the utility of the domain knowledge we have incorporated into the PLMs. It indicates that this specialized knowledge is particularly effective for enhancing the model’s capability to accurately predict outcomes for what are considered harder targets. This suggests that targeted interventions in the training process can yield substantial improvements in predictive accuracy.

### Contribution Shift

**Figure 4:**
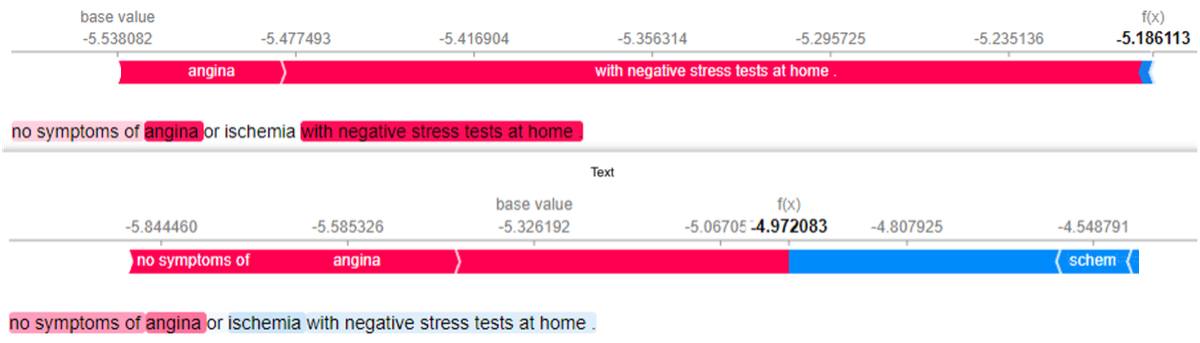
Contribution shift analysis of ClinicalBERT (top) and DAKI-ClinicalBERT (bottom). Darker shades of pink indicate a positive contribution and the shades of blue indicate a negative contribution to the target, that is, coronary heart disease. DAKI: Diverse Adapters for Knowledge Integration.

In the end, we would like to understand how the injected knowledge affects PLMs in the specific task. We use the SHapley Additive exPlanations (SHAP) tool, a game theoretic approach to explaining the output of any machine learning model [30], to explain the results. We take one of the hardest targets, that is, coronary heart disease, as an example and investigate the contribution distribution of tokens from ClinicalBERT and DAKI-ClinicalBERT, as shown in Figure 4. Red means positive contribution (ie, predicting the comment to be coronary heart disease among all the targets in this case), and blue means negative contribution. The *f(x)* is the model’s score for this observation, where a higher score leads the model to predict the specific class. Essentially, we observe that with the external knowledge, the DAKI-ClinicalBERT model is more sensitive to the tokens and their contribution to the prediction, compared with ClinicalBERT that treats the tokens almost equally. Such contribution shift indicates that the injected knowledge helps PLMs capture the semantics of text at the token level.

## Discussion

### Principal Findings

Interpretability is a major issue in machine learning, especially in the clinical setting. The reasons are 2-fold. First, it is essential for physicians to understand how a model is making its predictions in order to trust and effectively use the model. This is particularly important in the medical field because the consequences of incorrect predictions can be severe. Second, machine learning techniques, especially deep learning models, are hard to interpret, which makes it difficult for physicians to identify potential biases or errors in the model. To improve the interpretability in the application of machine learning to the clinical setting, we consider constructing a health knowledge graph so that the models are used responsibly and that the consequences of incorrect predictions are minimized.

In recent years, there has been a surge of interest in creating and using external health knowledge graphs to enhance the domain adaptation and interpretability of PLMs. An optimal health knowledge graph can be used for a variety of purposes, such as, (1) for research purposes, they could be used to represent the relationships between different medical conditions, treatments, and patient characteristics that a physician considers when deciding on a course of treatment for a patient and this could help to clarify the reasoning behind the decision and identify any factors that may have influenced the decision; (2) for analysis purposes, they could help to identify patterns and factors in physicians’ decision-making process, which can be important for improving the quality and efficiency of hospital care; and (3) for practical purposes, such graphs could support clinical decision-making by providing physicians with information and guidance to help them make informed decisions about patient care.

Nevertheless, traditional biomedical knowledge graphs, including the UMLS [31], the Systematized Nomenclature of Medicine Clinical Terms (SNOMED CT) [5], the Human Phenotype Ontology (HPO) [32], etc, mostly consist of biomedical concepts and their relationships, along with textual descriptions, and can struggle to fulfill the third purpose, that is, clinical practice. The reason lies in their limited capacity to incorporate practical physician knowledge, which is crucial for clinical applications.

Physician knowledge is a key item of interest for inclusion in health knowledge graphs, as mining a health knowledge graph (as opposed to manual construction) provides the potential for discovering latent clinical knowledge that may not be self-evident. Such items can be found within physician reasoning behind assigning a diagnosis, as such diagnoses are typically made based on an application of the individual physician’s knowledge. As physician reasoning is primarily not encoded in structured data forms, we must instead turn to NLP techniques, for example, the PLMs, on clinical narratives, which can include symptom descriptions, reasons for diagnosis, patient activities, and patient histories with the aim of helping physicians express a holistic picture of the patient [8].

As a preliminary step, we aim to explore and model the thought process and decision-making in the clinic by capturing physician reasoning. With the experiment of text-based diagnosis prediction, we believe that foundational PLMs are capable of capturing physician knowledge given relatively high performance in top-k (k=1,3,5,10) returned results, especially when compared with random chance.

Moreover, by injecting external domain knowledge from 3 disparate sources (ie, the UMLS Metathesaurus, the Wikipedia articles, and the semantic groupings) into the PLMs through adapters, we show that the models’ performance gets consistently improved with an increased level of interpretability. Essentially, the framework’s flexibility enables us to investigate and interpret what external domain knowledge is activated and how it contributes to the model in capturing physician reasoning.

The transferability of the knowledge adapters is also notably highlighted. As demonstrated in Table 1, DAKI-BERT’s performance, on par with ClinicalBERT, underscores the adapters’ transferability, given that DAKI-BERT achieves comparable performance even without using extensive and confidential clinical text corpora. This transferability implies the potential for infusing heterogeneous knowledge while honoring institutional boundaries; institutions can create their adapters using proprietary data and knowledge, thereby sharing only the foundational models and not the institution-specific adapters. This aspect warrants further exploration in future studies.

As for future work, we would further investigate the impact of different sources of knowledge over individual diagnosis, that is, how external knowledge affects the judgments over the 50 diagnoses. We would also explore and incorporate other sources of knowledge such as the ontological structure of the target diagnoses. By combining the internal knowledge within the EHRs and the external knowledge accumulated throughout knowledge bases under a unified framework, we would improve the interpretability of machine learning models in the clinical scenario and facilitate the construction of a health knowledge graph eventually.

Although the experiments demonstrate the effectiveness of our method, there are still some limitations that can be improved. First, the impact of knowledge adapters over different clinically relevant tasks remains unclear as only one task is considered in this work. Second, the range of external knowledge is a bit limited, for example, the inherent ontological structure of the targets is not leveraged, as mentioned above. Third, there is a lack of clinical explanation for the observations at an individual level, for example, why these knowledge adapters are most useful for “normocytic anemia.” We will try to fix these issues in future work.

### Conclusions

This study serves as a preliminary exploration of capturing physician reasoning. By predicting patients’ diagnoses based on physician comments, we aim to explore physician knowledge and the way they make judgments about the patients. We propose to inject domain knowledge from disparate sources into PLMs through adapters under the DAKI framework, enhancing their representation capability on clinical text. The experimental results demonstrate that capturing physician knowledge is feasible through the encoding of clinical text using PLMs, the representation capability and interpretability of which can be further improved when equipped with external domain knowledge. Notably, the transferability of the knowledge adapters, exemplified by comparable performance between DAKI-BERT and ClinicalBERT without access to extensive clinical corpora, underscores the potential for scalable and versatile applications across various institutional contexts and knowledge domains.

## Appendix

Multimedia Appendix 1 Activation levels of the adapters placed at different layers of BERT (left) and ClinicalBERT (right), respectively.

Multimedia Appendix 2 Greatest impact observed among the least successful targets.

## Abbreviations

ClinicalBERT: clinical bidirectional encoder representations from transformer
CTRL: knowledge controller
CUI: concept unique identifiers
DAKI: Diverse Adapters for Knowledge Integration
DS: disease adapter
HPO: Human Phenotype Ontology
IRP: impression/report/plan
KG: knowledge graph adapter
LoRA: low-rank
MIMIC-III: Medical Information Mart for Intensive Care III
NLP: natural language processing
PHI: protected health information
PLM: pretrained language model
SG: semantic grouping adapter
SHAP: SHapley Additive exPlanations
SNOMED CT: Systematized Medical Nomenclature for Medicine–Clinical Terminology
UMLS: Unified Medical Language System

## Data Availability

The Mayo Clinic IRP data set used in this study is not publicly available due to privacy risks and is not available by request. The source code of DAKI is available in the GitHub repository [6].
