# Supplementary figures and images for: Enhancing Clinical Relevance of Pretrained Language Models Through Integration of External Knowledge: Case Study on Cardiovascular Diagnosis From Electronic Health Records
Source: JMIR AI. 2024 Aug 6;3:e56932. doi: 10.2196/56932 (PMC11336492; doi:10.2196/56932)

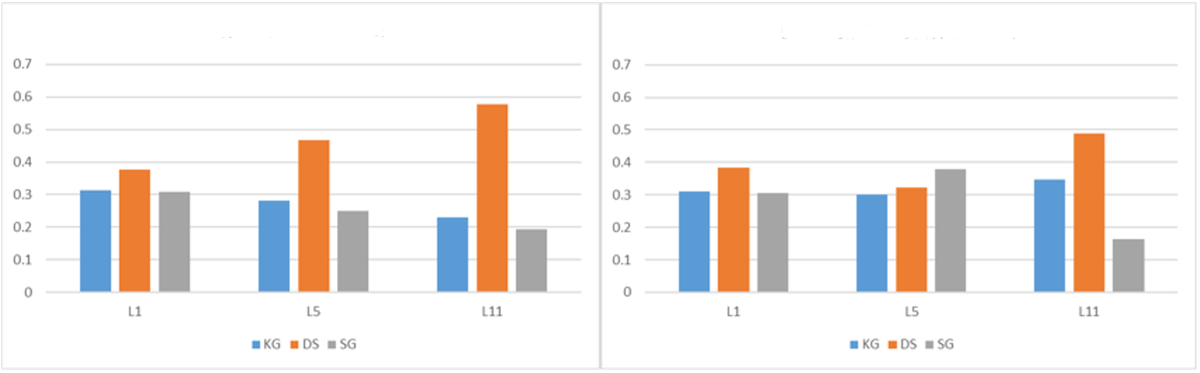

Supplement: Multimedia Appendix 1 [file ai_v3i1e56932_app1.png]

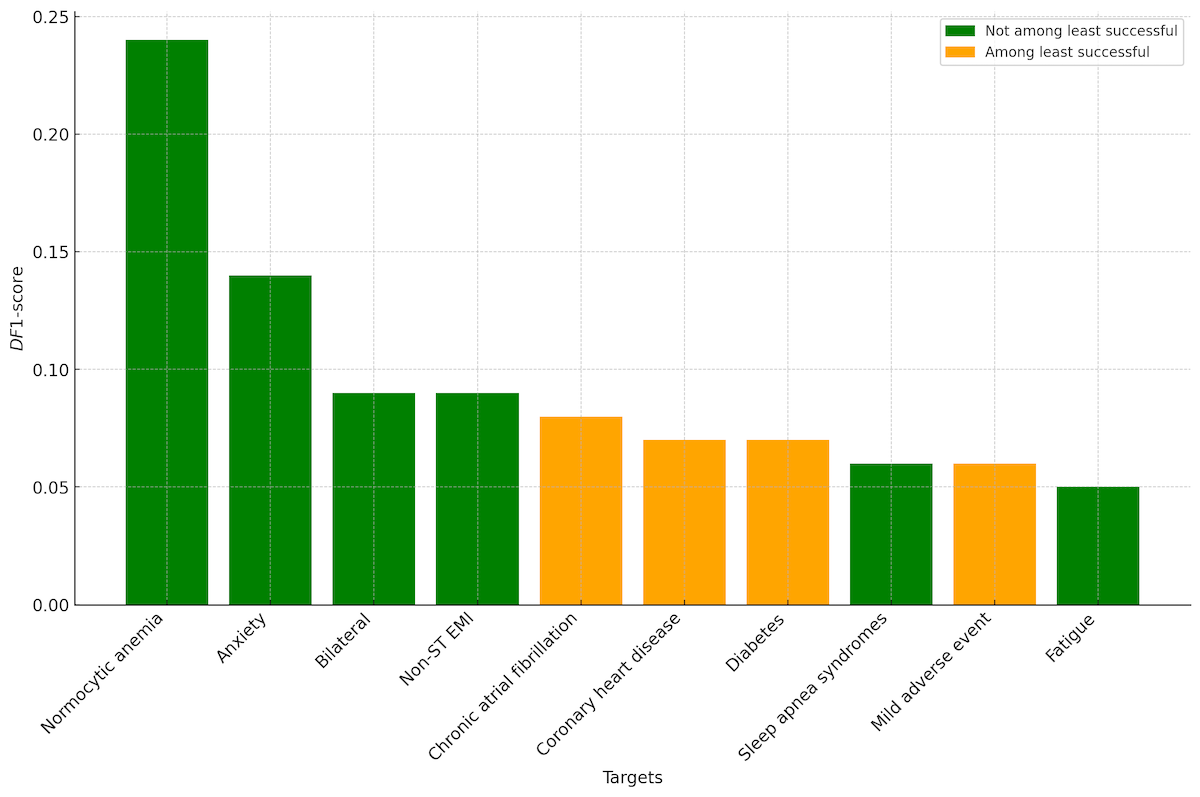

Supplement: Multimedia Appendix 2 [file ai_v3i1e56932_app2.png]
